# Performance of NEWS, qSOFA, and SIRS Scores for Assessing Mortality, Early Bacterial Infection, and Admission to ICU in COVID-19 Patients in the Emergency Department

**DOI:** 10.3389/fmed.2022.779516

**Published:** 2022-03-02

**Authors:** Julio Alencar, Luz Marina Gómez Gómez, Andre Lazzeri Cortez, Heraldo Possolo de Souza, Anna Sara Levin, Matias Chiarastelli Salomão

**Affiliations:** ^1^Departamento de Emergência Médica, Hospital das Clínicas, Faculdade de Medicina, Universidade de São Paulo, São Paulo, Brazil; ^2^Departamento de Moléstias Infecciosas e Parasitárias do Hospital das Clínicas da Faculdade de Medicina da Universidade de São Paulo, São Paulo, Brazil; ^3^Departamento de Emergência Médica, Faculdade de Medicina, Universidade de São Paulo, São Paulo, Brazil; ^4^Departamento de Moléstias Infecciosas e Parasitárias, Faculdade de Medicina, Universidade de São Paulo, São Paulo, Brazil

**Keywords:** COVID-19, sepsis, NEWS, qSOFA (quick sequential organ failure assessment), SIRS (for Systemic Inflammatory Response Syndrome), scores, prognosis, emergency

## Abstract

SARS-CoV-2 infection has a wide spectrum of presentations, from asymptomatic to pneumonia and sepsis. Risk scores have been used as triggers for protocols that combine several interventions for early management of sepsis. This study tested the accuracy of the score SIRS, qSOFA, and NEWS in predicting outcomes, including mortality and bacterial infection, in patients admitted to the emergency department (ED) during the COVID-19 pandemic. We described 2,473 cases of COVID-19 admitted to the ED of the largest referral hospital for severe COVID-19 in Brazil during the pandemic. SIRS, qSOFA and NEWS scores showed a poor performance as prognostic scores. However, NEWS score had a high sensitivity to predict in-hospital death (0.851), early bacterial infection (0.851), and ICU admission (0.868), suggesting that it may be a good screening tool for severe cases of COVID-19, despite its low specificity.

## Introduction

SARS-CoV-2 infection has a wide spectrum of presentations, from asymptomatic to severe cases of viral pneumonia and Acute Respiratory Distress Syndrome (1). Considering the pathophysiology and the clinical manifestations, some COVID-19 patients meet the definition of sepsis, described as an unregulated inflammatory host response to infection that results in organ failure and risk of death (2, 3).

This concept of sepsis is recent and was updated after a better understanding of pathophysiological events (4). In a consensus definition from 1991, sepsis was defined as a systemic inflammatory response (SIRS—Systemic Inflammatory Response Syndrome) caused by infection (5, 6). The diagnosis of sepsis was made in patients with suspected or confirmed infection and two of four criteria: abnormalities in body temperature, tachypnea, tachycardia and leukocytosis (6). More recently, a new consensus, Sepsis-3, defines sepsis as organ dysfunction, represented as at least 2 points in the Sequential Organ Failure Assessment (SOFA) score in patients with suspected or confirmed infection (3). In the Emergency Department (ED), the use of a sepsis-related organ failure prediction tool (qSOFA) can help identify patients at high risk of death (3). Moreover, authors have compared the accuracy of scores based on physical examination for diagnosing sepsis in patients admitted to the ED with suspected or confirmed infection, and the NEWS (National Early Warning Score) score has been shown superior to SIRS and qSOFA (7).

These three tools have been used as triggers for protocols that combine several interventions for early management of sepsis, including the use of antibiotics (8). Although there is still controversy about how quickly antibiotics should be administered to septic patients in general (9), COVID-19 is a viral disease without indication for antibiotic treatment (10), and there is concern that the use of antibiotics may exacerbate antimicrobial resistance without a clinical benefit (11).

Thus, we designed a study to test the accuracy of the scores SIRS, qSOFA, and NEWS in predicting outcomes, including mortality and bacterial infection, in patients admitted to the ED during the COVID-19 pandemic.

## Methods

### Study Design and Population

We conducted a retrospective single center cohort study from March to August 2020 at the ED in Hospital das Clínicas, in São Paulo, Brazil. This is an academic tertiary-care hospital affiliated to São Paulo University with 2,200 beds, comprising five institutes and two auxiliary hospitals. In March 2020, the main institute was converted to a COVID-19–only facility, dedicating 900 beds to the care of infected patients. Admissions to the COVID-19 Institute were centrally managed by the Regulatory Central of the State of São Paulo, and severely ill patients are preferably referred to the hospital

We included all consecutive adult patients (≥ 18 years) with confirmed COVID-19, defined as at least one positive result using reverse transcriptase-polymerase chain reaction (Rt-PCR) obtained from nasopharyngeal swabs or bronchial secretions (12).

We excluded patients for whom we could not calculate scores due missing data. Patient data were collected through electronic medical records, and a database was built using REDCap software (13).

We applied risk assessment scores according to patients' admission variables. The positive qSOFA cutoff was 2 or greater (3), NEWS score was classified into low risk (1–3 points) and high risk (four or more points) of sepsis (7), and the positive cutoff for SIRS was 2 or greater (5).

Besides the SIRS, qSOFA and NEWS variables, we also collected data on demographics (age, sex), clinical history (previous diagnoses and medications, time of symptoms on admission, physical examination, supplemental oxygen), laboratory tests routinely collected on admission (complete blood count, D-dimer, C-reactive protein, urea, creatinine, fibrinogen, lactate), variables of SAPS3, treatment (antibiotics, anticoagulants and corticosteroids), and outcomes (length of hospital stay, dialyses, invasive mechanical ventilation and in-hospital mortality). We considered with severe COVID-19, patients who had SpO2 < 90% on room air, clinical signs of pneumonia, or a respiratory rate >30 breaths/min (10).

The primary outcome was in-hospital mortality within 30 days after admission. Secondary outcomes were admission to intensive care unit (ICU) within 7 days from admission, and early bacterial infection confirmed by bacterial growth in culture.

We defined as early bacterial infection any positive culture of blood, urine or tracheal secretions in the first 7 days of hospitalization. We considered contaminants the coagulase-negative *Staphylococci, Corynebacterium species, Bacillus* spp. other than *Bacillus anthracis, Cutibacterium acnes, Micrococcus* spp., *viridans* group *streptococci, and Clostridium perfringens* (14) if isolated in only one culture of the patient. The contaminants were excluded.

All patients received standard care, according to the institutional protocol. In the emergency department, this included oxygen supplementation, dexamethasone and antibiotics.

The study protocol was approved by the Local Ethics Committee (number: 3.990.817; CAAE: 30417520.0.0000.0068), which waived the need for written informed consent. We adhered to Transparent Reporting of a Multivariable Prediction for Individual Prognosis or Diagnosis (TRIPOD) guidelines (15).

### Statistical Analysis

Mean, standard deviation (SD), median, and interquartile range (IQR) were used for descriptive statistics according to variable distribution.

Model predictive performance was assessed with the area under the receiver operating characteristics curve (AUROC). Clinical utility was analyzed using sensitivity, specificity, positive predictive values (PPV), negative predictive values (NPV), positive likelihood ratio, negative likelihood ratio, and precision recall curves. Confidence intervals (95%) were calculated after 1,000 bootstrap re-samples (16–19).

SIRS, qSOFA and NEWS's variables were submitted to bivariate analysis and factors with statistical significance (*p* < 0.05) were submitted to logistic regression using multivariate analysis by calculating the Lassos lambda coefficient for the outcomes of in-hospital death, ICU admission, and early bacterial infection.

A Bonferroni correction was used to account for multiple comparisons across the pre-specified outcomes and subgroup analyses.

All statistical analyses were performed using the software R version 3.6.2.

## Results

**Figure 1:**
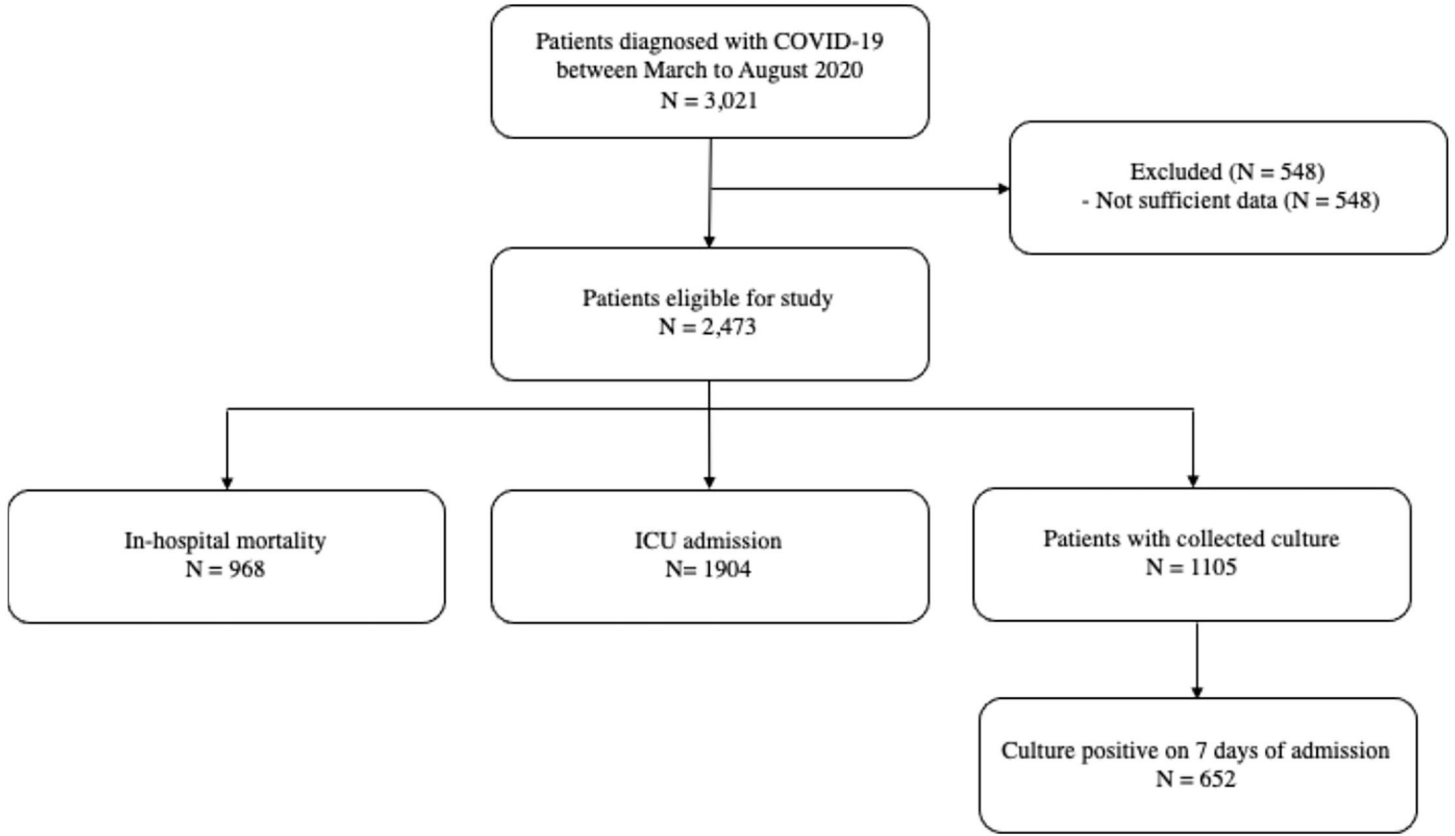
Patients flow.

A total of 3,021 patients diagnosed with COVID-19 in the Emergency Department were included in the study, of which 2,473 patients had enough data to calculate the scores. To analyze the predictive power of the three scores, data from these 2,473 individuals were used (Figure 1).

The median age of patients was 61.6 years, 57% were male, and the median length of hospital stay was 14 days. The median SAPS3 was 65, and the median time between onset of symptoms of COVID-19 and hospitalization was 8 days. A total of 1,904 patients (77%) required ICU admission. In-hospital mortality was 39% (968 patients) (Tables 1, 2). Cultures collected within the first 7 days of hospitalization were available for 1,190 patients, and 684 (62%) of these patients had an infectious agent isolated. The most commonly isolated agents were *Staphylococcus aureus* (112 isolates), *Candida albicans* (109 isolates), *Pseudomonas aeruginosa* (78 isolates), and *Acinetobacter baumannii* (69 isolates) (Table 3). The most isolated agents, considering only blood cultures, were *Staphylococcus aureus* (67 isolates), *Enterococcus* spp. (50 isolates), *Klebsiella pneumoniae* (48 isolates), *Pseudomonas aeruginosa* (34 isolates), *Acinetobacter baumannii* (30 isolates), *Candida albicans* (19 isolates), and other *Candida* species (23 isolates).

**Table 1:** Characteristics of patients on Emergency Department admission.

	**All patients** **(2,473)**			**Died in** **hospital (968)**			**Survivors** **(1,505)**				**All patients** **with cultures** **(1,105)**			**Patients with** **positive cultures** **(652)**			**Patients with** **negative cultures** **(453)**			
	**Median**	**Interquartile** **Interval**		**Median**	**Interquartile** **Interval**		**Median**	**Interquartile** **Interval**		***P*-value**	**Median**	**Interquartile** **Interval**		**Median**	**Interquartile** **Interval**		**Median**	**Interquartile** **Interval**		***P*-value**
Age	61.6	49.1	71.3	62.5	52.5	70.1	62.5	51	70.7	0.73	62.5	51.8	70.5	62.5	52.5	70.1	62.5	51	70.7	0.73
Hospital length of stay (days)	14	8	23	25	18	37	18	11	27	<0.01	21	13	32	25	18	37	18	11	27	<0.01
**Characteristics on admission**																				
Duration of Symptoms on Admission (days)	8	5	11	7	4	10	8	5	12	<0.01	8	5	11	7	4	10	8	5	12	<0.01
Temperature (°C)	36.1	36	37	36.3	36	37	36.2	36	37	0.76	36.2	36	37	36.3	36	37	36.2	36	37	0.76
Heart rate (bpm)	88	77	100	89	78	100	90	78	102	0.19	90	78	101	89	78	100	90	78	102	0.19
Respiratory Rate (ipm)	24	20	28	25	20	30	24	20	30	0.22	24	20	30	25	20	30	24	20	30	0.22
Systolic blood Pressure (mmHg)	122	110	139	120	109	137	120	105	137	0.38	120	107	137	120	109	137	120	105	137	0.38
SpO2 (%)	94	91	96	93	90	96	94	91	97	0.01	94	91	96	93	90	96	94	91	97	0.01
SAPS3	65	53	77	66	54.25	77	69	58	78.5	0.01	68	56	78	66	54.25	77	69	58	78.5	0.01
BMI	26.4	23.4	31.6	25.8	22.9	30.4	26.65	23.5	32	0.04	26.2	23.4	31.3	25.8	22.9	30.4	26.65	23.5	32	0.04
**Blood tests collected up to 72 h after admission**																				
Leukocytes (X 10^3^/μL)	9.06	6.27	12.84	9.17	5.96	13.67	10	7	15	<0.01	9.75	6.60	14.24	9.17	5.96	13.67	9.91	7.04	14.60	<0.01
Neutrophils (X 10^3^ / μL)	7.48	4.85	11	7.8	5	11.85	8.49	6	12.98	0.01	8.26	5.32	12.50	7.80	4.82	11.85	8.49	5.75	12.98	0.01
Lymphocytes (X 10^3^/μL)	0.85	0.56	1.22	0.71	0.48	1	0.81	0.52	1	0.02	0.78	0.50	1.14	0.71	0.48	1.08	0.81	0.52	1.19	0.02
CRP (mg/L)	128.5	63.7	236.4	168.55	88.58	271	169.2	80.8	269.3	0.61	169.2	84.2	270.8	168.55	89	271	169.2	80.8	269.3	0.61
LDH (UI/L)	436	316.5	593	495	378	631	501	376	678.5	0.40	498	377	656.5	495	378	631	501	376	678.5	0.40
D-Dimer (ng/mL)	1,631	878	5,030	1,697	940	5,286	2,954	1,198.5	7233.5	<0.01	2,241	1093.5	6,749	1,697	940	5,286	2,954	1198.5	7233.5	<0.01
Fibrinogen (mg/dL)	538	410	664	525	389	684	551	410	664	0.53	551	403	664	525	389	684	551	410	664	0.53
Lactate (mg/dL)	13	10	18	14	10	19	14	11	18	0.68	14	10.75	18	14	10	19	14	11	18	0.68

**Table 2:** Characteristics of patients on Emergency Department admission and outcomes.

	**All patients**		**Died in hospital (968)**		**Patients with positive cultures (652)**	
	**N (2,473)**	**%**	**N (968)**	**%**	**N (652)**	**%**
Sex (Male) *N*, %	1,412	57%	608	43%	400	61%
Comorbidities
Chronic kidney disease (Dialysis) *N*, %	659	27%	492	75%	282	59%
Cardiovascular disease *N*, %	460	19%	202	44%	114	57%
Hypertension *N*, %	1,445	59%	633	44%	430	62%
COPD *N*, %	166	7%	83	50%	53	64%
Asthma *N*, %	101	4%	26	26%	22	63%
Renal failure (dialysis) *N*, %	86	4%	43	50%	25	63%
Renal failure *N*, %	226	9%	111	49%	63	56%
Liver disease *N*, %	76	3%	38	50%	20	63%
Stroke *N*, %	182	7%	89	49%	51	59%
Dementia *N*, %	74	3%	42	57%	11	69%
Rheumatologic disease *N*, %	58	2%	15	26%	20	71%
Hematological disease *N*, %	176	9%	68	39%	58	60%
Psychiatric disease *N*, %	81	4%	24	30%	19	61%
Solid organ transplant *N*, %	70	9%	29	41%	14	44%
Obesity *N*, %	354	14%	95	27%	105	65%
Diabetes *N*, %	947	38%	428	45%	302	64%
Dyslipidemia *N*, %	144	18%	53	37%	32	47%
Cancer *N*, %	231	10%	134	58%	56	51%
Immunodeficiency *N*, %	44	4%	28	64%	13	42%
HIV/Aids *N*, %	21	1%	11	52%	6	50%
Hypothyroidism *N*, %	178	21%	74	42%	49	52%
Smoker *N*, %	167	7%	84	50%	56	58%
Alcoholism *N*, %	101	9%	38	38%	30	59%
Drug user *N*, %	23	3%	7	30%	7	58%
Other comorbidities *N*, %	373	24%	166	45%	104	59%
Symptoms on Admission
Dyspnea *N*, %	1,862	75%	750	40%	532	62%
Cough	1,664	68%	630	38%	433	59%
Sputum *N*, %	119	7%	40	34%	37	73%
Tiredness *N*, %	619	25%	208	34%	167	64%
New confusion *N*, %	149	6%	66	44%	35	69%
Life support
ICU *N*, %	1,904	77%	927	49%	637	61%
Mechanical Ventilation *N*, %	1,491	65%	878	59%	575	62%
Vasoactive drugs *N*, %	1,455	65%	881	61%	563	60%
Oxygen therapy *N*, %	2,307	95%	967	42%	669	62%
ECMO *N*, %	11	0%	9	82%	6	60%
Anticoagulant *N*, %	2,416	98%	948	39%	678	62%
Antiplaquet *N*, %	485	20%	191	39%	127	56%
Corticosteroid use *N*, %	1,695	69%	771	46%	544	62%
Use of immunosuppressants *N*, %	82	3%	31	38%	20	48%
Antibiotic *N*, %	2,291	93%	935	41%	661	61%
Antifungal *N*, %	242	10%	139	57%	118	61%
ACEi *N*, %	370	15%	74	20%	71	50%

**Table 3:** Bacterial infections.

**Isolate**	**Frequency**
**Early bacterial infection (culture positive on first 7 days of admission)**	
Other non-fermenting gram negative bacilli	13
*Acinetobacter baumannii* complex	69
Others	9
Anaerobes	4
Other *Candida* spp.	17
*Candida glabrata*	39
*Candida albicans*	109
*Candida tropicalis*	46
Other Enterobacterales	15
Complexo M. tuberculosis	10
Other Enterobacterales	4
Coagulase-negative *Staphylococcus*	60
*Streptococcus* spp.	6
*Serratia marcescens*	9
*Staphylococcus aureus*	112
*Escherichia coli*	36
*Klebsiella pneumoniae*	45
*Aspergillus* spp.	2
*Burkholderia* spp.	2
*Proteus* spp.	4
*Enterobacter cloacae* complex	10
*Pseudomonas aeruginosa*	78
*Stenotrophomonas maltophilia*	15

At admission, 1,364 (55%) had positive SIRS, 820 (33%) had positive qSOFA, and 2005 (81%) had high risk NEWS. In-hospital mortality frequency based on these cutoffs were: 629 (46%) for SIRS; 265 (32%) for qSOFA, and 859 (43%) for NEWS. The frequency of patients with early bacterial infection based on the cut-offs were: 423 (62%) for SIRS; 211 (66%) for qSOFA; and 582 (61%) for NEWS (Table 4).

**Table 4:** Scores SIRS, qSOFA and NEWS at admission and outcomes in patients COVID-19.

	**Patients**	**SIRS** **>** **2**		**qSOFA** **>** **2**		**NEWS** **>** **4**	
	***N***	***N***	**%**	***N***	**%**	***N***	**%**
Died in hospital	968	629	46%	265	32%	859	43%
Positive culture	652	423	62%	211	67%	582	61%

### Prediction of Mortality

The AUROC for each score to predict mortality was: 0.58 for SIRS, 0.55 for qSOFA, and 0.56 for NEWS. After corrections, only AUROC values for SIRS and qSOFA were considered statistically different (*p* = 0.003).

**Figure 2:**
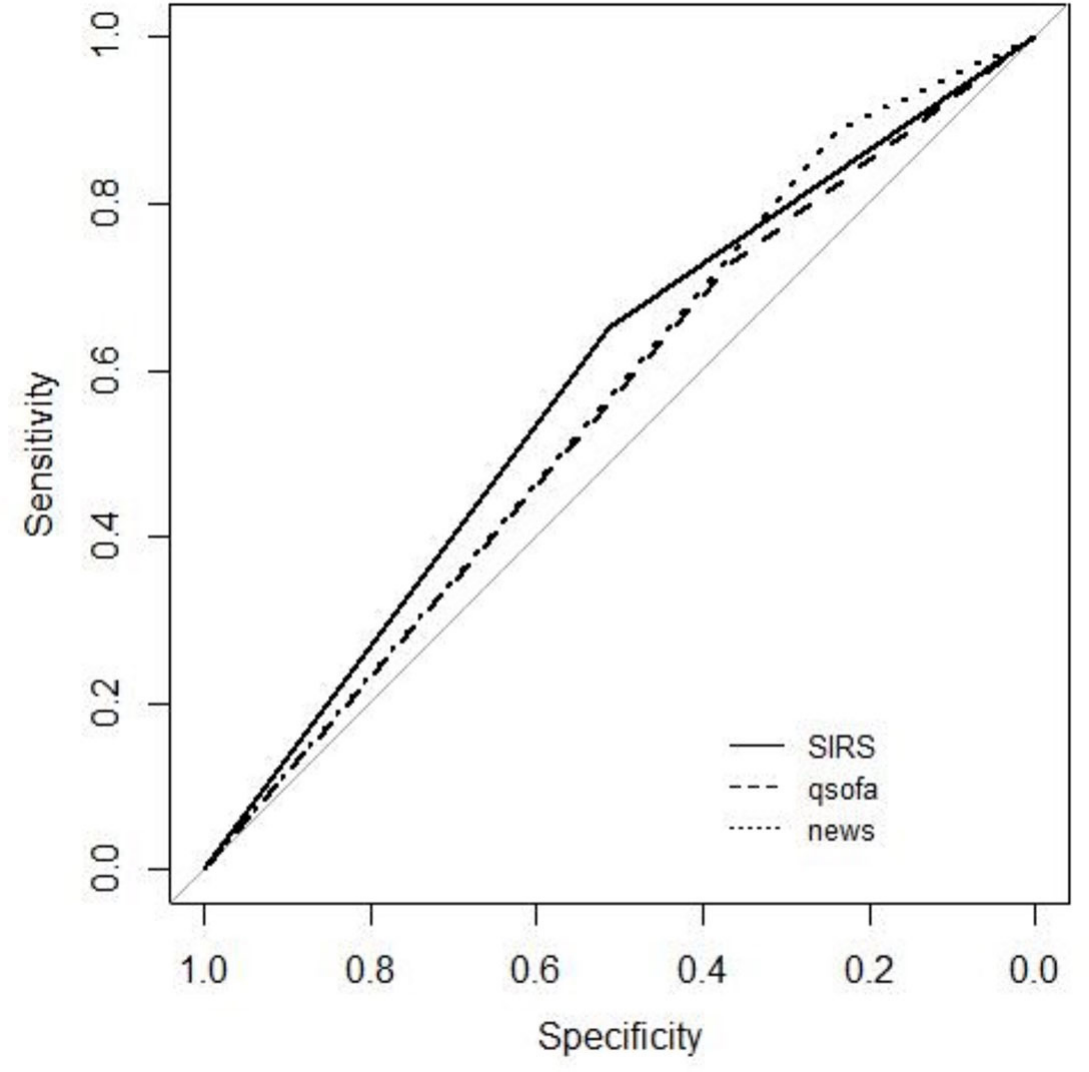
ROC curves for mortality.

We found higher sensitivity for NEWS 0.89 (CI 95% 0.87–0.91) and its NPV was 0.77 (CI 95% 0.73–0.80). However, NEWS had a lower specificity, 0.24 (CI 95% 0.22–0.26) and lower PPV 0.43 (CI 95% 0.42–0.44) (Table 5, Figure 2).

**Table 5:** Area under Receiver Operator Curves (AUROC) for mortality and early bacterial infection for SIRS > 2, qSOFA > 2, and NEWS > 4.

	**AUC**	**CI 95%**		***P-*value**	**Sensitivity**	**CI 95%**		**Specificity**	**CI 95%**		**NPV**	**CI 95%**		**PPV**	**CI 95%**	
**AUROC for mortality prediction for SIRS** **>** **2, qSOFA** **>** **2, and NEWS** **>** **4**																
SIRS	0.58	0.56	0.6	0.003^*^	0.65	0.61	0.68	0.51	0.49	0.54	0.69	0.67	0.71	0.46	0.44	0.48
qSOFA	0.55	0.53	0.57	0.08^***^	0.27	0.25	0.30	0.63	0.61	0.66	0.57	0.56	0.59	0.32	0.30	0.35
NEWS	0.56	0.55	0.58	0.09^**^	0.89	0.87	0.91	0.24	0.22	0.26	0.77	0.73	0.80	0.43	0.42	0.44
**AUROC for early culture positivity prediction for SIRS** **>** **2, qSOFA** **>** **2, and NEWS** **>** **4**																
SIRS	0.50	0.47	0.53	0.11*	0.61	0.58	0.65	0.37	0.33	0.42	0.38	0.34	0.41	0.62	0.59	0.64
qSOFA	0.53	0.50	0.56	0.49^***^	0.30	0.27	0.34	0.75	0.71	0.79	0.40	0.38	0.42	0.67	0.62	0.71
NEWS	0.52	0.50	0.54	0.24^**^	0.85	0.82	0.88	0.11	0.79	0.14	0.31	0.24	0.38	0.61	0.60	0.62

* *p-value comparison between SIRS and qSOFA*.

** *p-value comparison between SIRS and NEWS*.

*** *p-value comparison between qsofa and NEWS*.

### Prediction of Early Bacterial Infection

**Figure 3:**
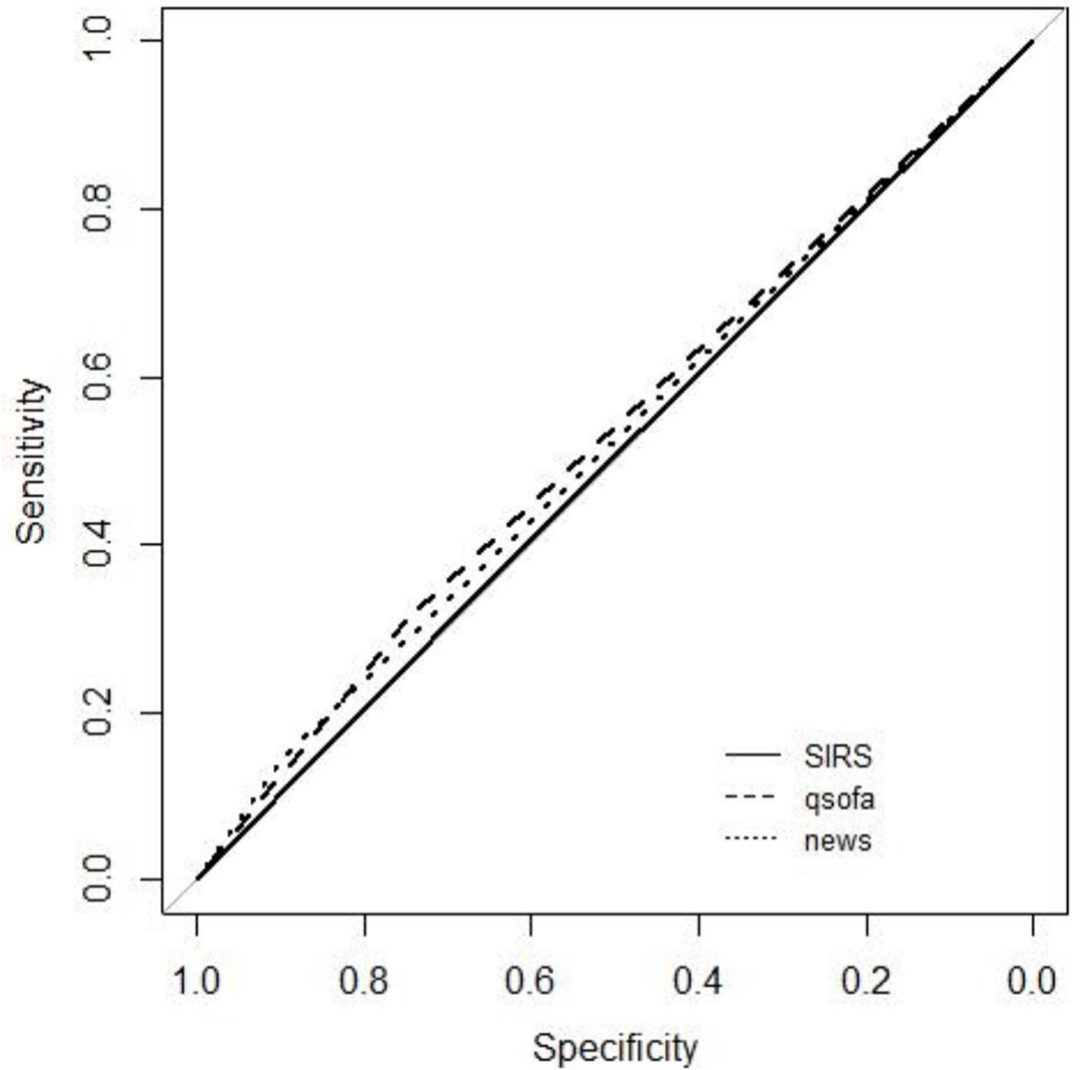
ROC curve for culture positivity.

There was no difference between the AUROC of the three scores to predict bacterial infection, with poor performance for the three. The NEWS score presented the best sensitivity [0.85 (CI 95% 0.82–0.88)], and qSOFA the best specificity [0.75 (CI 95% 0.71–0.79)] (Table 5, Figure 3).

### Prediction of ICU Admission

**Figure 4:**
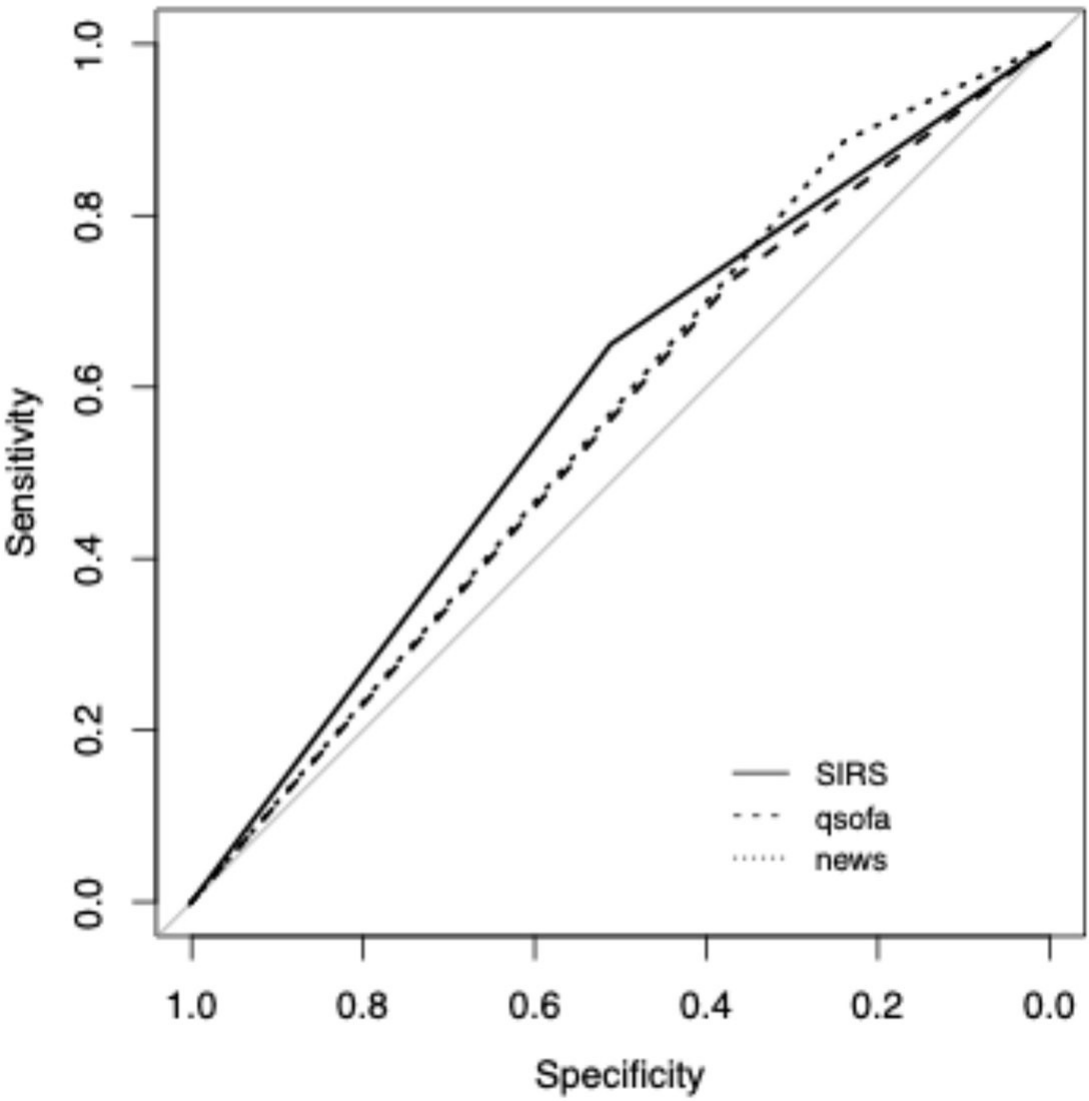
ROC curve for ICU admission.

There was also no difference between the AUROC of the three scores. The NEWS score demonstrated the best sensitivity [0.87, CI 95% (0.85; 0.88)], and SIRS [0.62, CI 95% (0.58; 0.66)] the best specificity (Figure 4).

### Factors Associated With Mortality, Admission to the ICU, and Early Bacterial Infection

The factors associated with in-hospital death were: use of steroids, cancer, male sex, and immunosuppression. Protective factors were: use of ACEi, rheumatologic disease, and hematologic disease (Table 6).

**Table 6:** Bivariate and multivariate analysis for In-hospital mortality, early bacterial infection, and ICU hospitalization in COVID-19 patients in the Emergency Department.

	**In-hospital mortality**					**Early Bacterial Infection**					**ICU hospitalization**				
	**RR**	***P*-value**	**CI 95%**		**Lassos lambda coefficient**	**RR**	***P*-value**	**CI 95%**		**Lassos lambda coefficient**	**RR**	***P*-value**	**CI 95%**		**Lassos lambda coefficient**
Age	1.05	<0.001	1.04	1.05	0.03	1.00	0.89	0.99	1.01		1.02	<0.001	1.01	1.02	0.01
Length of stay	0.99	0.04	0.99	1.00		0.98	<0.001	0.97	0.98		1.13	<0.001	1.12	1.15	
Time of symptoms on admission	1.00	0.73	0.98	1.01		1.05	<0.001	1.02	1.07	0.02	1.02	0.01	1.01	1.04	0.00
Temperature on admission	0.83	<0.001	0.76	0.90	−0.09	0.98	0.73	0.86	1.11		1.07	0.19	0.97	1.19	
Heart rate on admission	1.01	0.00	1.00	1.01	0.01	1.00	0.32	1.00	1.01		1.01	<0.001	1.01	1.02	0.00
Respiratory rate on admission	1.01	0.07	1.00	1.02		0.99	0.34	0.98	1.01		1.08	<0.001	1.06	1.10	0.04
Systolic blood pressure on admission	0.99	<0.001	0.99	0.99	0.00	1.00	0.44	0.99	1.00		0.99	0.00	0.99	1.00	−0.01
SpO2	0.96	<0.001	0.95	0.98	−0.02	1.02	0.04	1.00	1.04	0.01	0.96	<0.001	0.94	0.98	0.01
SAPS3	1.06	<0.001	1.05	1.07	0.05	1.01	0.01	1.00	1.02	0.01	1.10	0.05	1.01	1.21	
BMI on admission	0.98	<0.001	0.97	0.99	−0.01	1.01	0.26	1.00	1.02		1.00	0.93	0.99	1.01	
Leukocytes in the first 72 h	1.05	<0.001	1.03	1.06	0.00	1.01	0.10	1.00	1.03		1.14	<0.001	1.11	1.17	−0.01
Neutrophils in the first 72 h	1.10	<0.001	1.08	1.12	0.02	1.03	0.02	1.00	1.05	0.01	1.21	<0.001	1.18	1.25	0.12
Lymphocytes in the first 72 h	1.00	0.61	1.00	1.01		1.00	0.75	0.98	1.02		1.00	0.72	1.00	1.02	
CRP in the first 72 h	1.00	<0.001	1.00	1.01	0.00	1.00	0.94	1.00	1.00		1.01	<0.001	1.01	1.01	0.00
LDH in the first 72 h	1.00	<0.001	1.00	1.00	0.00	1.00	0.10	1.00	1.00		1.01	<0.001	1.01	1.01	0.00
D dimer in the first 72 h	1.00	<0.001	1.00	1.00	0.00	1.00	0.12	1.00	1.00		1.00	<0.001	1.00	1.00	0.00
Fibrinogen in the first 72 h	1.00	0.05	1.00	1.00		1.00	0.46	1.00	1.00		1.00	0.01	1.00	1.00	0.00
Lactate in the first 72 h	1.05	<0.001	1.03	1.06	0.01	1.00	0.60	0.99	1.02		1.04	0.00	1.02	1.06	0.02
Dialysis	8.28	<0.001	6.76	10.18		0.83	0.13	0.65	1.06		15.54	<0.001	9.92	26.00	1.89
Cardiovascular disease	1.27	0.02	1.04	1.56		0.77	0.10	0.57	1.05		1.28	0.06	1.00	1.65	0.35
Hypertension	1.61	<0.001	1.36	1.90	0.00	1.00	1.00	0.78	1.29		1.62	<0.001	1.34	1.96	0.00
COPD	1.61	0.00	1.17	2.20	0.00	1.09	0.70	0.69	1.76		1.58	0.03	1.05	2.46	0.02
Asthma	0.53	0.01	0.33	0.82	0.00	1.04	0.91	0.53	2.15		0.42	<0.001	0.28	0.63	−0.40
Renal failure (dialysis)	1.58	0.04	1.03	2.44		1.03	0.94	0.54	2.01		1.56	0.13	0.90	2.90	
Renal failure	1.57	0.00	1.19	02.06	0.00	0.75	0.16	0.51	1.12		1.09	0.62	0.79	1.53	
Liver disease	1.58	0.05	1.00	2.50	0.00	1.03	0.94	0.50	2.18		0.83	0.49	0.50	1.43	
Stroke	1.54	0.01	1.14	02.08	0.00	0.89	0.61	0.57	1.40		0.90	0.57	0.64	1.29	
Dementia	2.09	0.00	1.31	3.35	0.00	1.36	0.57	0.49	4.34		0.51	0.01	0.32	0.83	−0.21
Rheumatologic disease	0.54	0.04	0.29	0.95	−0.25	1.55	0.30	0.70	3.78		0.94	0.83	0.52	1.78	
Hematological disease	0.75	0.07	0.54	1.03	−0.22	0.94	0.79	0.62	1.46		0.37	<0.001	0.25	0.56	−0.79
Psychiatric disease	0.54	0.01	0.33	0.87	0.00	0.96	0.91	0.46	2.05		0.60	0.04	0.37	0.98	0.00
Obesity	0.52	<0.001	0.41	0.67	0.00	1.18	0.34	0.84	1.69		1.48	0.01	1.12	2.00	0.08
Diabetes	1.50	<0.001	1.28	1.78	0.00	1.18	0.19	0.92	1.51		1.48	<0.001	1.22	1.82	
Dyslipidemia	1.00	1.00	0.69	1.45		0.37	<0.001	0.22	0.65	−0.72	1.36	0.18	0.88	2.19	
Cancer	2.24	<0.001	1.71	2.96	0.26	0.62	0.02	0.41	0.92	−0.41	0.53	<0.001	0.40	0.72	−0.41
Immunodeficiency	2.45	0.01	1.33	4.69	0.18	0.44	0.03	0.21	0.91	−0.22	8.67	0.03	1.87	154.28	0.19
HIV/Aids	1.72	0.22	0.72	4.14		0.61	0.40	0.19	1.97		0.75	0.54	0.30	2.10	
Hypothyroidism	1.26	0.18	0.90	1.76		0.47	0.00	0.29	0.77	0.04	1.14	0.53	0.77	1.71	
Smoker	1.63	0.00	1.19	2.23	0.00	0.82	0.37	0.54	1.26		3.20	<0.001	1.93	5.72	0.13
Alcoholism	0.82	0.36	0.54	1.25		0.92	0.79	0.52	1.68		0.79	0.36	0.48	1.35	
Drug user	0.76	0.55	0.29	1.81		0.67	0.50	0.21	2.30		0.86	0.75	0.35	2.40	
Other comorbidities	0.86	0.20	0.68	1.09		0.94	0.74	0.68	1.33		0.14	<0.001	0.09	0.21	
Dyspnea	1.21	0.05	1.00	1.47	0.00	1.10	0.53	0.82	1.46		1.88	<0.001	1.53	2.31	0.00
Cough	0.84	0.05	0.71	1.00	0.00	0.74	0.02	0.57	0.96	−0.22	1.10	0.34	0.90	1.34	
Sputum	0.97	0.86	0.65	1.42		1.68	0.11	0.91	3.27		0.64	0.02	0.44	0.95	0.00
Tiredness	0.73	0.00	0.60	0.88	−0.02	1.11	0.48	0.83	1.48		0.86	0.18	0.70	1.07	
New confusion	1.25	0.18	0.90	1.75		1.37	0.31	0.76	2.57		0.53	<0.001	0.38	0.76	−0.26
Oxygen therapy	93.09	<0.001	20.82	1639.71	0.00	0.29	0.11	0.04	1.09		31.83	<0.001	19.23	56.32	1.57
ECMO	7.05	0.01	1.81	46.30		0.92	0.90	0.26	3.63		637021.36	0.96	0.00	NA	
Antiplaquet	1.01	0.90	0.83	1.24		0.75	0.06	0.56	1.01		1.19	0.16	0.94	1.52	
Corticosteroid use	2.46	<0.001	02.04	2.97	0.48	0.93	0.62	0.68	1.25		4.31	<0.001	3.54	5.25	0.69
Use of immunossupressors	0.94	0.80	0.59	1.48		0.55	0.06	0.29	1.02		0.71	0.17	0.45	1.18	
Antibiotic	3.11	<0.001	2.14	4.65	0.00	0.35	0.03	0.12	0.85	−0.31	2.62	<0.001	1.91	3.56	0.00
Antifungal	2.28	<0.001	1.75	2.99	0.09	0.93	0.66	0.68	1.28		3.09	<0.001	2.03	4.92	0.00
ACEi	0.34	<0.001	0.26	0.44	−0.54	0.57	0.00	0.40	0.81	−0.44	0.93	0.60	0.72	1.22	

The factors associated with ICU admission were: dialysis, supplemental oxygen therapy, use of steroids, anticoagulation, cardiovascular disease, and immunosuppression (Table 6).

### Precision Recall

**Figure 5:**
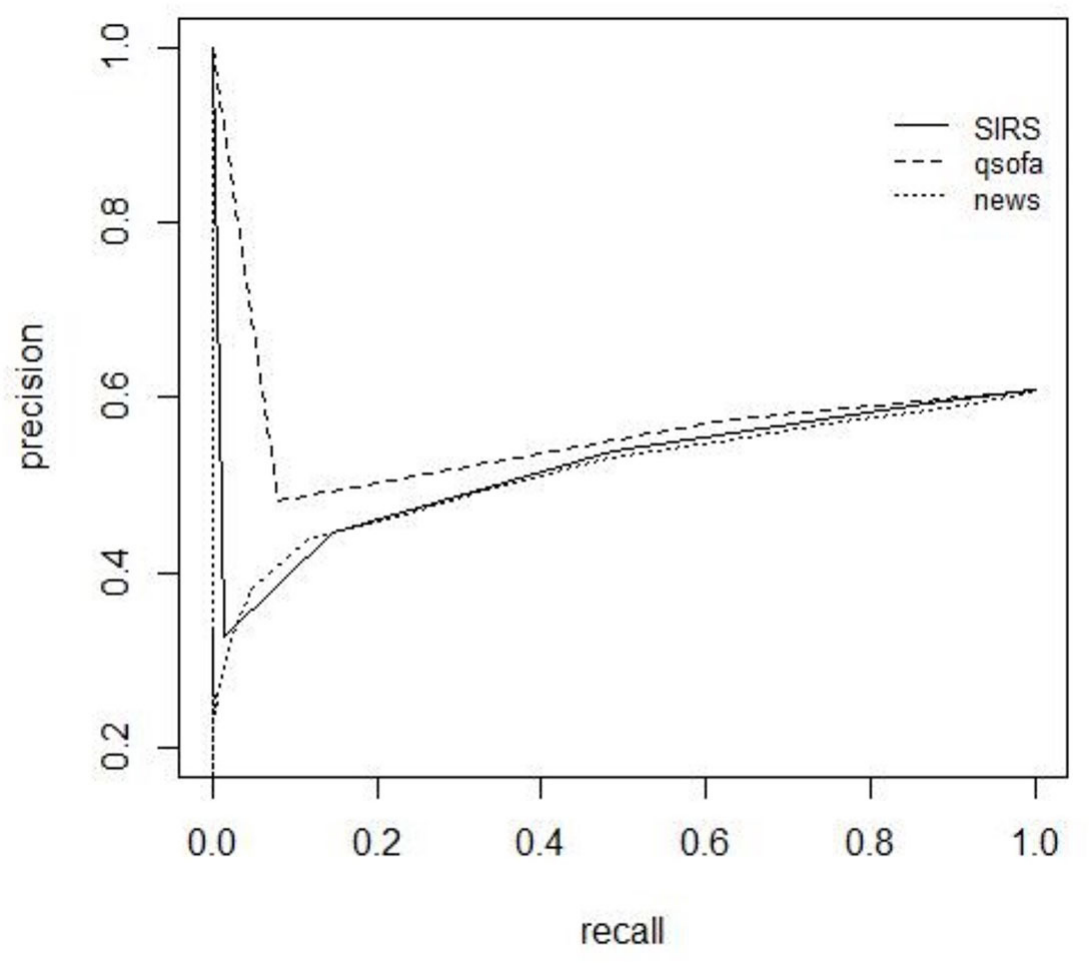
Precision recall (PR) curves for mortality.

**Figure 6:**
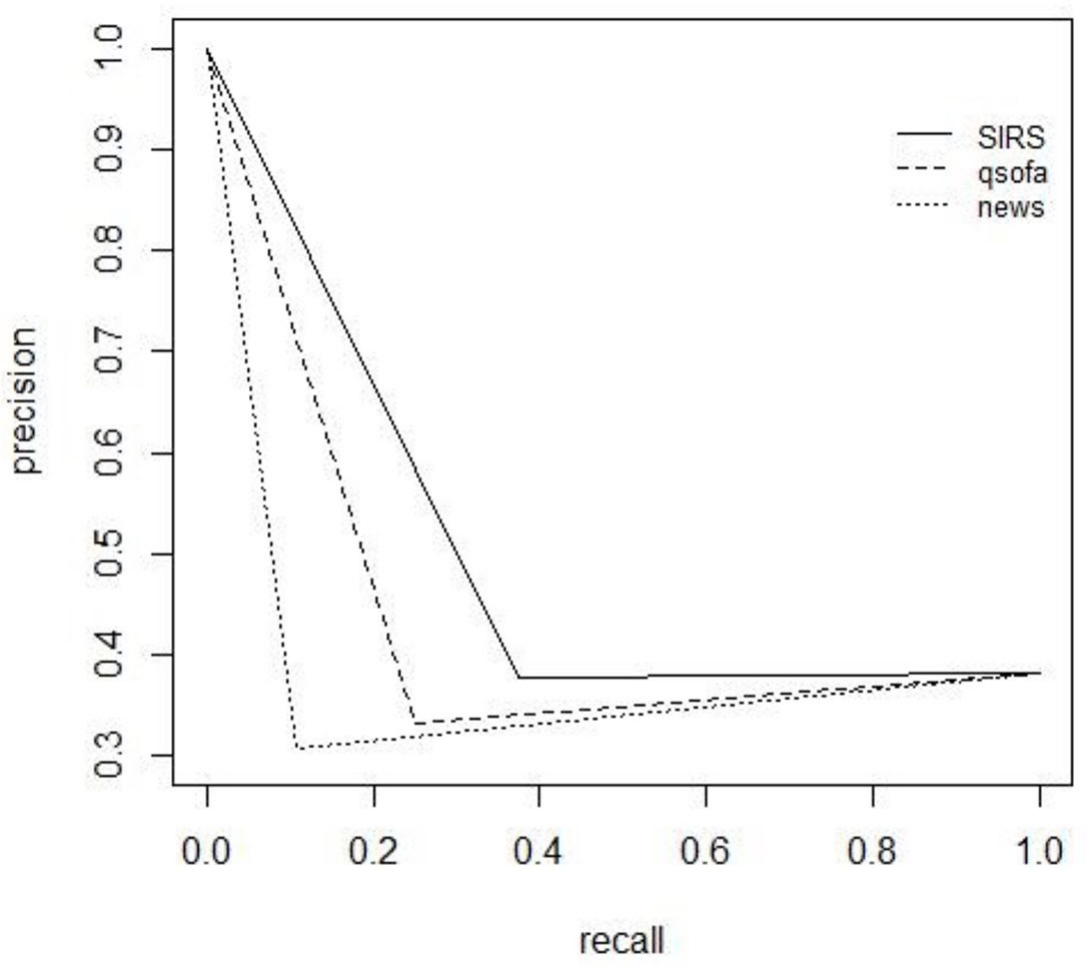
Precision recall (PR) curves for culture positivity.

All scores show low performance on precision-recall. They only presented a high recall value, but with small precision values. According to precision-recall, the score with the best performance is the qSOFA, which has the best specificity (Table 4). The scores also show a low performance to predict positive culture of patients with COVID-19. High precision values only are present with low recall (Figures 5, 6).

## Discussion

In this study we described 2.473 cases of COVID-19 admitted to the emergency department of a tertiary hospital during the pandemic, in order to evaluate the performance of SIRS, qSOFA and NEWS scores to predict in-hospital mortality, early bacterial infection, and ICU admission. Our findings suggest a poor performance of the 3 prognostic scores. However, they indicate a possible use of the NEWS as a screening tool for severe cases of COVID-19, given its high sensitivity to predict in-hospital death, early bacterial infection and ICU admission, despite its low specificity.

To our knowledge, this is the largest study to assess the performance of SIRS, qSOFA and NEWS scores in patients with COVID-19. Other authors have also evaluated prognostic scores to predict unfavorable outcomes for patients with COVID-19, but few have performed this assessment in the emergency department. Prognostic scores are tools that, in this context, help to make better-informed decisions (16, 17). In favor of the NEWS score, we must consider that this tool is already widely validated for the care of patients with sepsis. And, although not ideal, its high sensitivity allows NEWS to be used as a screening tool for cases that may progress badly during hospitalization. We also evaluated the performance of these tools to predict early bacterial infection, with similar results and NEWS also presented higher sensitivity than SIRS and qSOFA.

Our results are in agreement with the literature. The first study which systematically evaluated the use of NEWS2 for severe COVID-19 outcomes was carried out in five hospitals in the United Kingdom, one hospital in Norway, and two hospitals in Wuhan, China. Their results demonstrated a poor-to-moderate discrimination for 14-day ICU and death (AUC between 0.63 and 0.77 according to center) (20). Higher NEWS' cutoffs probably are better to predict COVID-19 outcomes. At Emergency Department, NEWS-2 score ≥ 6 at admission predicted severe disease with 80.0% sensitivity and 84.3% specificity (AUC 0.822, 95% CI 0.690–0.953), and was higher than qSOFA score ≥ 2 (AUC 0.624, 95% CI 0.446–0.810, *p* < 0.05) (21).

Although this study was conducted in an emergency department of a single center, this hospital was the main state referral for severe COVID-19. São Paulo has a population over 44 million, and 600 of the 6,000 critical COVID-19 care beds were located in this hospital. Because of this, our sample represents the selection of the most severe cases of the State of São Paulo, one of the world's epicenters of the pandemic at that time. This is evident when evaluating the median SAPS 3 value of 68 for patients admitted to the emergency department, which would have an expected mortality of 66.8% for patients seen in Latin America. We highlight that tools presented lower AUROCs than those found in some studies (12, 13, 16, 22, 23), mainly due to the lower specificity and PPV values. This may have happened because of the high severity of the cases. In a scenario with a higher prevalence of milder cases, there would be a better chance of detecting survivors, resulting in higher specificity and PPV values.

The incidence of early bacterial infection was high, 59% among those who collected cultures, and 26% among the 2,403 patients studied. This result is much higher than that found in other studies, 3–8% (24, 25). It would be expected that these infections had occurred later, but the median time of COVID-19 symptoms on admission was 8 days. This finding may be one of the factors related to the greater severity of our patients.

There were no factors strongly associated with early bacterial infection, but antibiotic use was associated with a reduced risk. This finding may be explained by the use of antibiotics resulting in negative cultures. Despite the high incidence of early bacterial infection, it is important to note that the use of antibiotics was not associated with lower risk of admission to the ICU or death. It was not possible to analyze the risk of developing infection by resistant bacteria in our study, but the indiscriminate use of antibiotics has been shown to be associated with the emergence of resistance, and the prescription of these drugs should be done cautiously and rationally.

Among the factors associated with in-hospital death, we found the use of steroids to be the most important factor. This may represent a bias as steroids are prescribed for severe COVID-19 as well as for comorbidities such as cancer and immunodeficiency. Paradoxically, rheumatologic disease and hematologic disease were not associated with death. The latter, it was not even a factor associated with admission to the ICU. These patients were prioritized for hospital care, which may have positively influenced the outcome, despite their potentially higher risk (26, 27). The use of ACEi was a protective factor against death in our study, as demonstrated by other authors (28, 29).

The most important factors associated with admission to the ICU admission were factors associated with the need for intensive support, such as dialysis, or the severity of COVID-19 (supplemental oxygen therapy, use of steroids and anticoagulation). The presence of cardiovascular disease and immunodeficiency were also factors associated with admission to the ICU. Factors not associated with hospitalization in ICU were: cancer, dementia, hematologic disease and asthma. Although not expected, patients with asthma had lower risk of hospitalization in ICU, as demonstrated in other studies (30–32).

This study has limitations. Data for this study were collected prospectively, but their analysis was performed later, and it was not possible to obtain retrospectively some data that were not collected initially. For instance, it was not possible to collect data on Glasgow Coma Scale for all patients, as this information was sometimes described as mental status alert, somnolent, and unconsciousness in the electronic medical record. We considered any positive culture as bacterial infection. It was not possible to evaluate the clinical features of the patients, so patients that were only colonized may have been considered as infected in our definition. We could not evaluate the antimicrobial resistance profiles in our study, so we could not analyze the impact of antibiotic use. This study was performed in a single-center, which is a limitation. However, this center was the reference hospital for severe cases of COVID-19 in the State of São Paulo, so we feel that it was broadly representative of the state which was hit hard by the pandemic. Our cases reflected the selection of the most severe cases in the state, actually representing a wider population than the study design would suggest, especially among critically ill patients in the emergency department.

In conclusion, for patients with severe COVID-19 admitted to the emergency department, SIRS or qSOFA did not perform well in predicting in-hospital mortality, early bacterial infection, or admission to the ICU. However, high sensitivity in predicting these three outcomes suggests that the NEWS score can be useful as a screening tool.

## Data Availability Statement

The raw data supporting the conclusions of this article will be made available by the authors, without undue reservation.

## Ethics Statement

The studies involving human participants were reviewed and approved by Hospital of Clinics, Faculty of Medicine, University of São Paulo, Brazil. Written informed consent for participation was not required for this study in accordance with the national legislation and the institutional requirements.

## Author Contributions

JA and MS conceptualized the project, analyzed the results, wrote the first draft, and wrote the final manuscript. LM performed the statistical analysis. AC collected data. HP and AL conceptualized the project and reviewed the manuscript. All authors contributed to the article and approved the submitted version.

## Funding

LM acknowledges the partial support of FAPESP (Fundação de Amparo à Pesquisa do Estado de São Paulo), grant 2019/23078-1. HP acknowledges the partial support of FAPESP, grants 2016/14566-4 and 2020/04738-8.

## Conflict of Interest

The authors declare that the research was conducted in the absence of any commercial or financial relationships that could be construed as a potential conflict of interest.

## Publisher's Note

All claims expressed in this article are solely those of the authors and do not necessarily represent those of their affiliated organizations, or those of the publisher, the editors and the reviewers. Any product that may be evaluated in this article, or claim that may be made by its manufacturer, is not guaranteed or endorsed by the publisher.

## Appendix

### HCFMUSP COVID-19 Study Group Authors

**Table:**

**Members**	**Name for publication**	**e-mail**	**ORCID-ID**
Amanda Montal	Amanda C. Montal	amanda.montal@hc.fm.usp.br	0000-0002-6478-6925
Anna Miethke Morais	Anna Miethke-Morais	anna.morais@hc.fm.usp.br	0000-0002-5077-4122
Beatriz Perondi	Beatriz Perondi	beatriz.perondi@hc.fm.usp.br	0000-0003-2280-642X
Carolina Carmo		carolina.carmo@hc.fm.usp.br	
Carolina dos Santos Lázari		carolina.lazari@hc.fm.usp.br	
Fabiane Yumi Ogihara Kawano		fabiane.kawano@hc.fm.usp.br	
Izabel Cristina Rios	Izabel Cristina Rios	izabel.rios@hc.fm.usp.br	0000-0003-0938-6459
Izabel Marcilio	Izabel Marcilio	izamarcilio@gmail.com	0000-0002-2914-6535
Juliana Carvalho Ferreira	Juliana C. ferreira	juliana.ferreira@hc.fm.usp.br	0000-0001-6548-1384
Rodrigo Antonio Brandão Neto		rodrigo.neto@hc.fm.usp.br	
Sabrina Ribeiro	Sabrina C. C. Ribeiro	sabrina.ribeiro@hc.fm.usp.br	0000-0002-1182-8415
Suze M. Jacon		suze.jacon@hc.fm.usp.br	
Leila Letaif	Leila Harima	leila.suemi@hc.fm.usp.br	0000-0003-0713-6560
Marcello Mihailenko Chaves Magri	Marcello M. C. Magri	marcello.magri@hc.fm.usp.br	
Marcelo Rocha	Marcelo C. Rocha	marcelo.rocha@hc.fm.usp.br	0000-0001-6821-2286
Maria Amélia de Jesus		maria.amelia@hc.fm.usp.br	0000-0001-8508-2612
Maria Cristina Peres Braido Francisco		maria.braido@hc.fm.usp.br	
Marjorie Fregonesi	Marjorie F. Silva	marjorie.silva@hc.fm.usp.br	
Maura Salaroli de Oliveira	Maura Salaroli Oliveira	maura.oliveira@hc.fm.usp.br	
Alberto José da Silva Duarte		alberto.duarte@hc.fm.usp.br	
Aluisio Segurado	Aluisio C. Segurado	segurado@usp.br	0000-0002-6311-8036
Carlos Carvalho		carlos.carvalho@hc.fm.usp.br	
Edivaldo Utiyama	Edivaldo M. Utiyama	edivaldo.utiyama@hc.fm.usp.br	0000-0002-8376-975X
Ésper Georges Kallas		esper.kallas@usp.br	
Tarcisio P. Barros Filho	Tarcisio E. P. Barros-Filho	tarcisio.barros@hc.fm.usp.br	0000-0002-7969-7845
Clarice Tanaka	Clarice Tanaka	clarice.tanaka@hc.fm.usp.br	0000-0003-3900-5944
Eloisa Bonfá	Eloisa Bonfa	eloisa.bonfa@hc.fm.usp.br	
Ester Sabino		sabinoec@gmail.com	
Silvia Figueiredo Costa	Silvia F. Costa	silviacosta@usp.br	
Solange Fusco	Solange R. G. Fusco	solange.fusco@hc.fm.usp.br	0000-0002-1243-1743
Thaís Guimarães	Thaís Guimarães	thais.guimaraes@hc.fm.usp.br	0000-0002-7282-5453

## References

[1] Zhu N Zhang D Wang W Li X Yang B Song J. A novel coronavirus from patients with pneumonia in China, 2019. N Engl J Med. (2020) 382:727–33. 10.1056/NEJMoa2001017 31978945 PMC7092803

[2] Merad M Martin JC. Pathological inflammation in patients with COVID-19: a key role for monocytes and macrophages. Nat Rev Immunol. (2020) 20:355–62. 10.1038/s41577-020-0331-4 32376901 PMC7201395

[3] Singer M Deutschman CS Seymour CW Shankar-Hari M Annane D Bauer M. The Third International Consensus definitions for sepsis and septic shock (Sepsis-3). JAMA. (2016) 315:801. 10.1001/jama.2016.0287 26903338 PMC4968574

[4] Shankar-Hari M Singer M Phillips G Levy M Rubenfeld G Angus D. Developing a new definition and assessing new clinical criteria for septic shock: for the Third International Consensus Definitions for Sepsis and Septic Shock (Sepsis-3). JAMA J Am Med Assoc. (2014) 312:775–87. 10.1001/jama.2016.0289 26903336 PMC4910392

[5] ACCP/SCCM Consensus Conference Committee Bone RC Balk RA Cerra FB Dellinger RP. Definitions for sepsis and organ failure and guidelines for the use of innovative therapies in sepsis. Chest. (1992) 101:1644–55. 10.1378/chest.101.6.1644 1303622

[6] Levy MM Fink MP Marshall JC Abraham E Angus D Cook D. International sepsis definitions conference. Crit Care Med. (2003) 31:1250–6. 10.1097/01.CCM.0000050454.01978.3B 12682500

[7] Usman OA Usman AA Ward MA. Comparison of SIRS, qSOFA, and NEWS for the early identification of sepsis in the Emergency Department. Am J Emerg Med. (2019) 37:1490–7. 10.1016/j.ajem.2018.10.058 30470600

[8] Baghdadi JD Brook RH Uslan DZ Needleman J Bell DS Cunningham WE. Association of a care bundle for early sepsis management with mortality among patients with hospital-onset or community-onset sepsis. JAMA Intern Med. (2020) 180:707. 10.1001/jamainternmed.2020.0183 32250412 PMC7136852

[9] Sterling SA Miller WR Pryor J Puskarich MA Jones AE. The impact of timing of antibiotics on outcomes in severe sepsis and septic shock. Crit Care Med. (2015) 43:1907–15. 10.1097/CCM.0000000000001142 26121073 PMC4597314

[10] World Health Organization. Clinical Management of COVID-19. Washigton, DC: WHO (2020).

[11] Ginsburg AS Klugman KP. COVID-19 pneumonia and the appropriate use of antibiotics. Lancet Glob Health. (2020) 8:e1453–4. 10.1016/S2214-109X(20)30444-7 33188730 PMC7833845

[12] Corman VM Landt O Kaiser M Molenkamp R Meijer A Chu DK. Detection of 2019 novel coronavirus (2019-nCoV) by real-time RT-PCR. Euro Surveill. (2020) 25:2000045. 10.2807/1560-7917.ES.2020.25.3.2000045 31992387 PMC6988269

[13] Harris PA Taylor R Minor BL Elliott V Fernandez M O'Neal L. The REDCap consortium: building an international community of software platform partners. J Biomed Inform. (2019) 95:103208. 10.1016/j.jbi.2019.103208 31078660 PMC7254481

[14] Doern GV Carroll KC Diekema DJ Garey KW Rupp ME Weinstein MP. Practical guidance for clinical microbiology laboratories: a comprehensive update on the problem of blood culture contamination and a discussion of methods for addressing the problem. Clin Microbiol Rev 33. (2019) e00009–19. 10.1128/CMR.00009-19 31666280 PMC6822992

[15] Collins GS Reitsma JB Altman DG Moons KG. Transparent reporting of a multivariable prediction model for individual prognosis or diagnosis (TRIPOD): the TRIPOD statement. BMJ. (2015) 350:g7594. 10.1136/bmj.g7594 25569120

[16] Brown LD Cai TT DasGupta A. Interval estimation for a binomial proportion. Stat Sci. (2001) 16:101–17. 10.1214/ss/1009213286

[17] Mercaldo ND Lau KF Zhou XH. Confidence intervals for predictive values with an emphasis to case-control studies. Stat Med. (2007) 26:2170–83. 10.1002/sim.2677 16927452

[18] Boyd K Eng KH Page CD. Area under the precision-recall curve: point estimates and confidence intervals. In: Blockeel H, Kersting K, Nijssen S, Železný F, editors. Machine Learning and Knowledge Discovery in Databases. ECML PKDD 2013. Lecture Notes in Computer Science, vol. 8190. Berlin; Heidelberg: Springer (2013).

[19] Davis J Goadrich M. (2006). The relationship between precision-recall and ROC curves. In: Proceedings of the 23^rd^ International Conference on Machine Learning. Pittsburgh, PA (2006). 10.1145/1143844.1143874

[20] Carr E Bendayan R Bean D Stammers M Wang W Zhang H. Evaluation and improvement of the National Early Warning Score (NEWS2) for COVID-19: a multi-hospital study. BMC Med. (2021) 19:23. 10.1101/2020.04.24.20078006 33472631 PMC7817348

[21] Myrstad M Ihle-Hansen H Tveita AA Andersen EL Nygård S Tveit A. National Early Warning Score 2 (NEWS2) on admission predicts severe disease and in-hospital mortality from Covid-19 - a prospective cohort study. Scand J Trauma Resusc Emerg Med. (2020) 28:66. 10.1186/s13049-020-00764-3 32660623 PMC7356106

[22] Kostakis I Smith GB Prytherch D Meredith P Price C Chauhan A. The performance of the National Early Warning Score and National Early Warning Score 2 in hospitalised patients infected by the severe acute respiratory syndrome coronavirus 2 (SARS-CoV-2). Resuscitation. (2021) 159:150–7. 10.1016/j.resuscitation.2020.10.039 33176170 PMC7648887

[23] Jang JG Hur J Hong KS Lee W Ahn JH. Prognostic accuracy of the SIRS, qSOFA, and NEWS for early detection of clinical deterioration in SARS-CoV-2 infected patients. J Korean Med Sci. (2020) 35:e234. 10.3346/jkms.2020.35.e234 32597046 PMC7324266

[24] Vaughn VM Gandhi TN Petty LA Patel PK Prescott HC Malani AN. Empiric antibacterial therapy and community-onset bacterial coinfection in patients hospitalized with Coronavirus Disease 2019 (COVID-19): a multi-hospital cohort study. Clin Infect Dis. (2021) 72:e533–41. 10.1093/cid/ciaa1239 32820807 PMC7499526

[25] Langford BJ So M Raybardhan S Leung V Soucy JR Westwood D. Antibiotic prescribing in patients with COVID-19: rapid review and meta-analysis. Clin Microbiol Infect. (2021) 27:520–31. 10.1016/j.cmi.2020.12.018 33418017 PMC7785281

[26] Piñana JL Martino R García-García I Parody R Morales MD Benzo G. Risk factors and outcome of COVID-19 in patients with hematological malignancies. Exp Hematol Oncol. (2020) 9:21. 10.1186/s40164-020-00177-z 32864192 PMC7445734

[27] Yigenoglu TN Ata N Altuntas F Basci S Dal MS Korkmaz S. The outcome of COVID-19 in patients with hematological malignancy. J Med Virol. (2021) 93:1099–104. 10.1002/jmv.26404 32776581 PMC7436524

[28] Baral R Tsampasian V Debski M Moran B Garg P Clark A. Association between renin-angiotensin-aldosterone system inhibitors and clinical outcomes in patients with COVID-19: a systematic review and meta-analysis. JAMA Netw Open. (2021) 4:e213594. 10.1001/jamanetworkopen.2021.3594 33787911 PMC8013817

[29] Brandão Neto RA Marchini JF Marino LO Alencar JCG Lazar Neto F Ribeiro S. (2021). Correction: mortality and other outcomes of patients with coronavirus disease pneumonia admitted to the emergency department: a prospective observational Brazilian study. PLoS ONE. 16:e0248327. 10.1371/journal.pone.0248327 PMC7932119 33662001

[30] Yang JM Koh HY Moon SY Yoo IK Ha EK You S. Allergic disorders and susceptibility to and severity of COVID-19: a nationwide cohort study. J Allergy Clin Immunol. (2020) 146:790–8. 10.1016/j.jaci.2020.08.008 32810517 PMC7428784

[31] Choi YJ Park JY Lee HS Suh J Song JY Byun MK. Effect of asthma and asthma medication on the prognosis of patients with COVID-19. Eur Respir J. (2021) 57:2002226. 10.1183/13993003.02226-2020 32978309 PMC7518077

[32] Grandbastien M Piotin A Godet J Abessolo-Amougou I Ederlé C Enache I. SARS-CoV-2 pneumonia in hospitalized asthmatic patients did not induce severe exacerbation. J Allergy Clin Immunol Pract. (2020) 8:2600–7. 10.1016/j.jaip.2020.06.032 32603901 PMC7320869

